# The Effect of Stress-Reducing Interventions on Heart Rate Variability in Cardiovascular Disease: A Systematic Review and Meta-Analysis

**DOI:** 10.3390/life14060749

**Published:** 2024-06-12

**Authors:** Ouahiba El-Malahi, Darya Mohajeri, Alexander Bäuerle, Raluca Mincu, Korbinian Rothenaicher, Greta Ullrich, Christos Rammos, Martin Teufel, Tienush Rassaf, Julia Lortz

**Affiliations:** 1 Department of Cardiology and Vascular Medicine, West-German Heart and Vascular Center Essen, University of Duisburg-Essen, Hufelandstr. 55, 45147 Essen, Germany; 2 Clinic for Psychosomatic Medicine and Psychotherapy, LVR-University Hospital Essen, University of Duisburg-Essen, Virchowstr. 174, 45147 Essen, Germany; 3 Center for Translational Neuro- and Behavioral Sciences (C-TNBS), University of Duisburg-Essen, 45147 Essen, Germany

**Keywords:** cardiovascular disease, stress reduction, stress management, heart rate variability, cardiac rehabilitation

## Abstract

Stress is recognized as a significant trigger and exacerbator of various medical conditions, particularly in the field of cardiovascular disease (CVD). Given that heart rate variability (HRV) offers insight into the functioning of the autonomic nervous system and has been identified as a predictive factor for increased cardiovascular mortality, exploring the correlation between stress and HRV is pertinent. We systematically reviewed trials where researchers investigated the effects of stress-reducing interventions on biomarkers and time-domain/frequency-domain parameters of HRV in CVD. Eligible studies underwent meta-analysis utilizing a random-effects model. The meta-analysis showed overall beneficial effects of stress-reducing interventions on HRV for the standard deviation of Normal-to-Normal intervals (SDNN) in short-term and 24 h assessments, as well as for the low-frequency power (LF) in short-term assessment. Overall effect sizes were notably high and showed significant *p*-values (short-term SDNN: MD = 6.43, *p* = 0.01; 24 h SDNN: MD = 10.92, *p* = 0.004; short-term LF: MD = 160.11, *p* < 0.001). Our findings highlight the significant impact of stress-reducing interventions in modulating HRV by influencing short-term SDNN and LF parameters, as well as the 24 h assessment of SDNN. These results emphasize the importance of stress-reducing measures in lowering the risk of further progression in CVD and improving patient outcomes.

## 1. Introduction

Heart rate variability (HRV) serves as a valuable indicator of the fluctuation in time intervals between successive R-spikes of an electrocardiogram (ECG), reflecting the balance between the parasympathetic nervous system (PSNS) and the sympathetic nervous system (SNS). Physiologically, the sinoatrial node, responsible for cardiac pacemaking, is influenced by these neural structures, primarily governed by the SNS and the PSNS. Various factors such as temperature regulation, hormones, physical activity, and psychological stress can affect the autonomous nervous system and consequently impact HRV [1]. 

HRV can be measured using either time-domain or frequency-domain methods. The standard deviation of Normal-to-Normal intervals (SDNN) in the time-domain method provides an overall assessment of variability, while frequency-domain analysis involves dividing NN intervals into pre-defined frequency bands, such as high-frequency power (HF; 0.15–0.40 Hz) and low-frequency power (LF; 0.04–0.15 Hz). The ratio of LF to HF can indicate the balance of the autonomic nervous system, with a low LF/HF ratio suggesting PSNS dominance and a high ratio indicating SNS dominance [2]. 

Stress is a known contributor to the development and progression of various illness, particularly cardiovascular conditions. Studies show a positive correlation between stress and the development and progression of cardiovascular disease (CVD) [3,4]. Stress manifests through clinical observations like increased blink and breathing rates, as well as elevated blood pressure and heart rate. Laboratory measurements, including cortisol levels, c-reactive protein (CRP) and fibrinogen, also correlate with stress [5,6,7,8,9,10,11,12,13,14]. Additionally, psychometric questionnaires like the Perceived Stress Scale and the General Health Questionnaire provide insights into self-assessment of stress [15,16].

Numerous methods exist for stress reduction, including physical activities like yoga and tai chi, psychological techniques like mindfulness-based stress reduction and cognitive behavioral therapy and alternative therapies like acupuncture and massage. Practitioners of these interventions aim to alleviate stress by enhancing endogenous stress management, promoting relaxation and improving emotional well-being [17,18,19,20,21,22,23].

In this study, researchers sought to enlighten the relationship between interventions for stress reduction and the impact of these actions on HRV. It was felt that tracking these data could provide a monitoring tool for stress management among patients with CVD.

## 2. Materials and Methods

This systematic literature review and meta-analysis, registered on the International Prospective Register of Systematic Reviews (PROSPERO) under registration number CRD42023425081, adhered to the Preferred Reporting of Items for Systematic Reviews and Meta-Analyses (PRISMA) guidelines [24]. Given that the analyses utilized previously published data, ethical approval and written consent from participants were deemed unnecessary.

### 2.1. Search Strategy

We systematically searched PubMed, Embase and Cochrane Library for literature using free text and/or Medical Subject Headings (MeSH) or Emtree to search on Embase. In addition, already completed as well as ongoing studies were searched on ClinicalTrials.gov, German Clinical Trials Register (GermanCTR), International Clinical Trials Registry Platform (ICTRP) and International Standard Randomised Controlled Trial Number (ISRCTN) registry. No restrictions concerning language or publication date were set on all databases and clinical trial registers that were searched.

The following keywords were used for free text search: “major adverse cardiovascular event”, “myocardial infarction”, “coronary infarction”, “cardiac infarction”, “ischemic heart disease”, “heart failure”, stroke, “peripheral occlusive disease”, “coronary revascularization”, “cardiac arrhythmia”, “cardiovascular disease”, “coronary artery disease”, “acute coronary syndrome”, “cardiac arrest”, “heart arrest”, “heart attack”, “atrial fibrillation”, “stress management”, “stress reduction”, “yoga”, “biofeedback”, “behavioral therapy”, “acupuncture”, “mind-body”, “mindfulness-based”, “meditation”, “tai chi”, “heart rate variability”, “c-reactive protein”, “cortisol”, “fibrinogen”, “respiratory rate”, “blink rate”, “randomized controlled trial”, “randomized controlled study” and “random”.

Suitable MeSH were searched on the MeSH database of PubMed. Thus, the following MeSH were defined: “Cardiovascular Diseases”[Mesh], “Stroke”[Mesh], “Percutaneous Coronary Intervention”[Mesh], “Arrhythmias, Cardiac”[Mesh], “Atrial Fibrillation”[Mesh], “Yoga”[Mesh], “Biofeedback, Psychology”[Mesh], “Cognitive Behavioral Therapy”[Mesh], “Acupuncture Therapy”[Mesh], “Mind-Body Therapies”[Mesh], “Tai Ji”[Mesh], “C-Reactive Protein”[Mesh], “Hydrocortisone”[Mesh], “Fibrinogen”[Mesh], “Respiratory Rate”[Mesh] and “Blinking”[Mesh].

As search terms included multiple keywords (including MeSH or terms from Emtree), the operators “AND” as well as “OR” were used. Additionally, keywords that consist of at least two words were searched for using the phrase search. Subsequent to building the search term, the Polyglot Search Translator was used to convert the search term for the databases Cochrane Library and Embase [25]. Regarding Embase, all MeSH were manually searched in Emtree and replaced by suitable terms in the previously converted search term. The converted search term for the Cochrane Library was adjusted manually so that it only contains free text terms. Used search strings can be obtained from Table S1 of the Supplementary Materials.

### 2.2. Study Selection Process

During the selection process, EndNote (Version 20.5.0 for Windows, Clarivate Analytics, Philadelphia, PA, USA) was used as software for literature management. Initially, all identified publications and clinical trials with registered entries were imported into EndNote (Version 20.5.0 for Windows, Clarivate Analytics, Philadelphia, PA, USA) and checked for duplicates. Subsequently, all identified duplicates were removed and the systematic screening for eligibility of the remaining publication was started by reading the title and abstract. Afterwards, each screened publication was either excluded for a reason in the same step or checked for full text in a second step. Within the second step, the full text of the screened publication was read and either finally included or excluded in the systematic review. Exclusion reasons were documented in EndNote (Version 20.5.0) for each excluded publication separately during the title and abstract screening as well as during the full text screening process. 

Clinical trial register entries were firstly screened by reading all available information in the register entry before a decision was made to exclude this study or to proceed to a second screening step. Within the second screening step, study protocols and reports were searched for each study entering the clinical trial register number in Google. If no suitable protocols or reports were found, an e-mail was sent to the contact person named in the clinical trial register to request the results of the study. Discrepancies encountered during the literature search were deliberated between two researchers until a consensus was reached.

Studies fulfilling the following criteria were considered eligible: (i) adults (≥18 years old) after/with myocardial infarction (MI), congestive heart failure (CHF), coronary artery disease (CAD), peripheral occlusive disease, coronary revascularization, cardiac arrest, cardiac arrythmia or stroke as participants in the intervention and control group; (ii) acupuncture, biofeedback, cognitive behavioral therapy, meditation, mindfulness-based stress reduction, tai chi or yoga as intervention; (iii) control group receiving usual care or no intervention; (iv) intervention period ≥ 4 weeks; (v) at least one of the following outcome parameters was reported for the intervention and the control group: fibrinogen level, cortisol level, high-sensitivity CRP (hsCRP) level, CRP level, breathing rate, blink rate or at least one time-domain or frequency-domain parameter of HRV; (vi) study type was a randomized controlled trial (RCT). 

Studies were considered not eligible in cases for which at least one of the following criteria was met: (i) healthy participants in the intervention group and/or control group; (ii) physical activity interventions other than tai chi or yoga (e.g., resistance training, aerobic exercise training, Qigong) as well as acupressure or electro-acupuncture as intervention; (iii) education sessions, sham acupuncture, sham biofeedback or another sham treatment in the control group during the study period; (iv) outcomes of interest were not reported (v); case-control studies, letters, reviews, meta-analyses, animal studies and non-RCTs; (vi) studies without accessible abstract or full text (not extracted from clinical trial registers). 

### 2.3. Data Extraction

Data extraction was conducted in three parts. Within the first part, data were extracted for ongoing studies. For this, the following information was extracted from study protocols and/or clinical trial register entries of eligible ongoing studies: study name (official name), study identification number, study design, inclusion criteria for participating, interventions, outcome of interest, starting date and contact person. These data were recorded in a Microsoft Excel (Version 2304 for Windows, Microsoft Corporation, Redmond, United States) data sheet. Within the second part of the data-extraction process, the same data (as mentioned above) were recorded for eligible completed studies for which published results were either not found or not accessible. This information was extracted from clinical trial register entries and clinical trial protocols (if available) and were recorded in a similar Microsoft Excel (Version 2304 for Windows, Microsoft Corporation, Redmond, WA, USA) data sheet. Additionally, important notes related to requests via e-mail were recorded as well in a separate column in both data sheets. Data extraction for eligible studies with published results was conducted within the third part of the data-extraction process. For this, a third Microsoft Excel (Version 2304 for Windows, Microsoft Corporation, Redmond, WA, USA) data sheet was used in which the following data were recorded: last name of the first author, year of publication, study identification number, study information (study design, randomization method used, blinding, intention-to-treat-analysis), sample size (separately for the intervention and the control group), demographic data (mean age and gender distribution separately for intervention and control group), studied CVD (as defined in the inclusion criteria), other reported diseases involving the heart, criteria for inclusion and exclusion, information about the intervention (type, period, cycles), investigated outcome of interest (including assessment and analysis method), results related to the outcome of interest (pre- and post-interventional values) and shortened conclusion of the author. In addition, information related to requests or important information and date of extraction were recorded in separate columns in the Microsoft Excel data sheet.

### 2.4. Risk of Bias Assessment

The Risk-of-Bias 2 tool for RCTs was used for quality assessment regarding risk of bias [26]. Two researchers evaluated the risk of bias of all included studies independently from each other. Differences in quality assessment between these two researchers were discussed after the assessment for all included studies was completed. 

### 2.5. Data Synthesis

Pre- and post-interventional values for the intervention and the control group were summarized in Microsoft Excel for the following outcome parameters: fibrinogen, hsCRP, CRP, breathing rate, RR-Interval and SDNN as time-domain parameters of HRV, and total power (TP), LF, HF, LF in normal units (nLF), HF in normal units (nHF) and the LF/HF ratio as frequency-domain parameters of HRV [27]. Only values given as mean values with their standard deviation (SD) were considered eligible for data synthesis.

Outcome data that were only reported on a logarithmic scale were converted to values on a raw scale to avoid mixed data in the meta-analysis. For this, a formula presented in a previous study was used [28]. The calculation was done in Microsoft Excel (Version 2304 for Windows, Microsoft Corporation, Redmond, WA, USA) in several steps. Data that were expressed as geometric means on a logarithmic scale were not converted and were excluded from the meta-analysis. Converted values with extremely high SD compared to their mean values were also excluded.

### 2.6. Meta-Analysis

We conducted the meta-analysis for the parameter CRP, the time-domain parameter SDNN and the frequency-domain parameters TP, LF, HF and nHF using IBM SPSS Statistics (Version 29.0.0.0 for Windows, International Business Machines Corporation, Armonk, NY, USA). Meta-analysis of the time-domain parameter SDNN was conducted for short-term as well as 24 h assessments separately. The parameters TP, LF, HF and nHF of HRV were only available for short-term measurements. For all analyses, post-interventional values of the intervention group and of the control group were used. Differential values were not considered for meta-analysis. We conducted all meta-analyses using a random effect model and the mean difference (MD) as the effect size. For heterogeneity assessment, the I^2^ statistic was chosen for all analyses. For the short-term parameter SDNN, subgroup analyses were conducted concerning the intervention period and the total size of analyzed participants, also using IBM SPSS Statistics (Version 29.0.0.0 for Windows, International Business Machines Corporation, Armonk, NY, USA). A subgroup analysis related to the type of intervention or disease concerning the short-term parameter SDNN was not conducted, as the intervention type was equal in all four studies [29,30,31,32] and three of four studies investigated the same main CVD [29,31,32]. For the other outcome parameters, subgroup analyses were not performed, as we had determined to conduct such analyses only when data from a minimum of four studies were available.

For the sensitivity analysis, the effect size standard mean difference (SMD) was chosen. All outcome parameters were analyzed within a sensitivity analysis using IBM SPSS Statistics (Version 29.0.0.0 for Windows, International Business Machines Corporation, Armonk, NY, USA). 

## 3. Results

### 3.1. Study Selection and Characteristics

**Figure 1:**
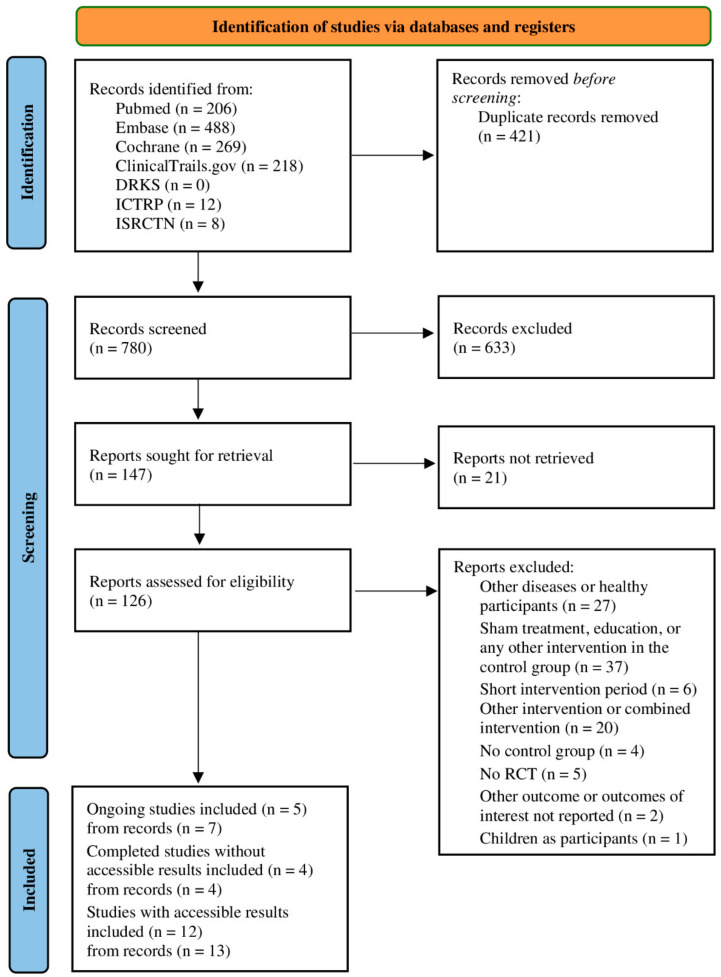
PRISMA flow chart showing details of the processes related to the identification, screening and selection process. RCT = randomized controlled trial.

After removing duplicates, 780 publications were identified from databases and clinical trial registers within this systematic literature research. After the full text screening process, 21 studies (extracted from 24 records) were included: 5 ongoing studies (extracted from 7 records), 4 completed studies without accessible/published results (extracted from 4 records) and 12 completed studies with published results (extracted from 13 records). The PRISMA flow chart in Figure 1 shows the details of the study selection process [24]. The characteristics of the included studies with published results are presented in Table 1.

Characteristics of the included ongoing studies [43,44,45,46,47,48,49] and the studies without accessible/published results [50,51,52,53,54] are shown in Tables S2 and S3 of the Supplementary Materials.

All studies included in the analysis were RCTs, where participants were randomly assigned to either an intervention group receiving a specific intervention or a control group receiving usual care/no intervention [29,30,31,32,33,34,35,36,37,39,40,41,42]. Fibrinogen levels were reported in one study [33], while CRP, hsCRP and breathing rate were reported in two studies each [29,30,33,34,35,38]. At least one time-domain or frequency-domain parameter of HRV as an outcome was reported in nine studies [29,30,31,32,36,37,39,40,41,42]. Of these, one study reported daytime and nighttime values of HRV extracted from a 24 h ECG recording device [36]. Two other studies reported 24 h values of HRV also extracted from a 24 h ECG recording device [30,41,42]. Short-term values of HRV were reported by six studies in total, as one study reported 24 h values as well as short-term values of HRV [29,30,31,32,37,40]. In one study, the values of change during deep-breathing sessions were reported for one parameter of HRV [39]. None of the included studies with accessible results reported cortisol or blink rate as an outcome parameter. 

### 3.2. Study Selection and Characteristics

**Figure 2:**
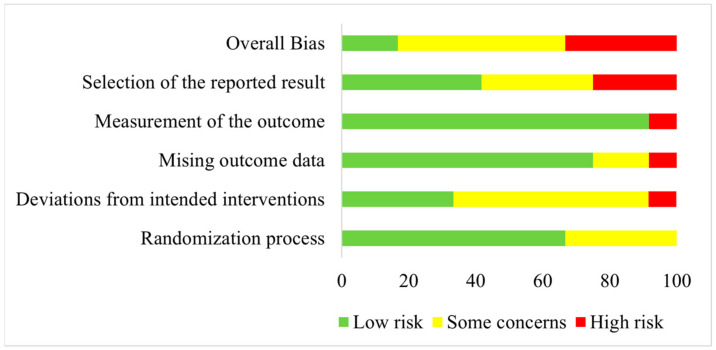
Risk-of-bias graph illustrating the results of quality assessment for randomization process, deviations from intended interventions, missing outcome data, measurement of the outcome, selection of the reported results and overall bias for all included studies.

Quality assessment regarding risk of bias was conducted for all included studies listed in Table 1. Results are shown as a risk-of-bias graph in Figure 2. 

An overall high risk of bias was assessed for four studies [31,33,36,40]. Of these, one study was rated as having a high risk of bias concerning deviations from the intended intervention and missing outcome data [33]. Concerning the selection of the reported results, three studies were judged to have a high risk of bias [31,36,40]. Of these, one study were also rated with a high risk of bias related to the measurement of outcome [40]. All other included studies were either rated as having a low risk of bias or with some concerns for the five investigated domains [29,30,32,34,35,37,38,39,41,42]. 

### 3.3. Meta-Analysis

Nine studies were assessed as eligible and included for meta-analyses of the different outcome parameters of interest [29,30,31,32,34,35,37,40,41,42]. Exclusion from meta-analysis was not performed due to the risk of bias assessment. The outcome parameter fibrinogen was excluded from the meta-analysis since only one study reported post-interventional values of this parameter [33]. The parameter RR-Interval was also not considered in the meta-analysis, as the two studies reporting this parameter were not comparable to each other [39,41,42]. One study reported the change in this parameter during forced deep breathing [39] and the other study reported values assessed using a 24 h ECG device [41,42]. Breathing rate as an outcome parameter was also not analyzed within a meta-analysis since the two studies reporting this parameter were not comparable to each other [29,30]. One study used “breaths/min” [29], whereas the other study used “L/min” as units to report the breathing rate [30]. The outcome parameter hsCRP was also not considered in a meta-analysis, as one of the two reporting studies reported the post-interventional values on a logarithmic scale as geometric mean values [33], whereas the second study used non-logarithmic and non-geometric mean values [38]. Within the meta-analysis of the parameters LF and HF, one eligible study was not considered since the SD, which was initially transformed from a logarithmic to a raw scale, was much higher in comparison to the mean value [30]. Concerning the parameters nHF and nLF, only nHF was analyzed within a meta-analysis since these two parameters are equivalent [55,56]. The LF/HF ratio was also not considered in meta-analysis since one study reported very high post-interventional values [29], and another study reported an extremely high SD in the control group in comparison to the mean value [37].

### 3.4. Outcome Parameters

#### 3.4.1. C-Reactive Protein (CRP)

Analysis of CRP was conducted with two eligible studies and 48 participants in both groups, each as shown in Figure S1 of the Supplementary Materials [34,35]. The overall effect size between the intervention and the control group was small (MD = 0.58) and not significant (*p* = 0.70). Heterogeneity was assessed as considerable as shown by the I^2^ value (I^2^ = 0.83). One study showed lower CRP levels in the intervention group compared to the control group after an intervention period of 12 weeks [34]. The other study reported lower CRP levels in the control group in comparison to the intervention group after 16 weeks [35]. In both studies, CHF patients were investigated either performing yoga [34] or tai chi [35] in the intervention group during the intervention period. 

#### 3.4.2. Standard Deviation of Normal-to-Normal Intervals (SDNN)

**Figure 3:**
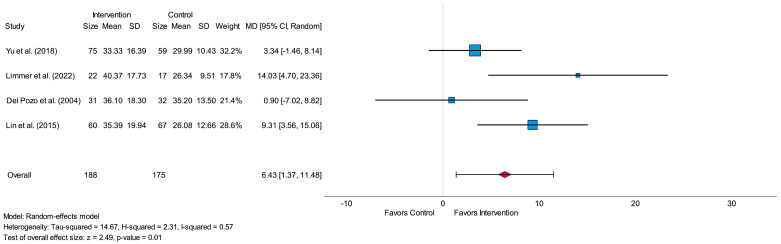
Forest plot of the time-domain parameter standard deviation of Normal-to-Normal intervals (SDNN) measured in milliseconds related to short-term measurement showing the effect of HRV-biofeedback in the intervention group compared to usual care/no intervention in the control group. SD = standard deviation, MD = mean difference, CI = confidence interval [29,30,31,32].

The meta-analysis of SDNN for short-term assessment was conducted with four eligible studies [29,30,31,32]. In total, 188 participants were analyzed in the intervention and 175 participants in the control group. All analyzed studies resulted in higher mean values in the intervention group in comparison to the control group after the intervention period [29,30,31,32]. The overall effect size between the intervention and the control group was high (MD = 6.43) and led to a significant value (*p* = 0.01), as shown in the forest plot in Figure 3. The I^2^ value (I^2^ = 0.57) led to moderate heterogeneity. In all four studies, HRV-biofeedback was investigated as a type of intervention [29,30,31,32]. Three studies focused on participants with CAD [29,31,32] and one study on participants after MI [30]. 

For 24 h assessment of SDNN, two studies were included in meta-analysis [30,41,42], in which 33 participants were analyzed in the intervention and 30 participants in the control group. The analysis across both studies showed a high value (MD = 10.92) with no heterogeneity (I^2^ = 0.00) and a *p*-value lower than 0.05 (*p* = 0.004), as shown in Figure S2 of the Supplementary Materials. One study focused on participants after MI [30] and the other study on CHF patients [41,42].

**Figure 4:**
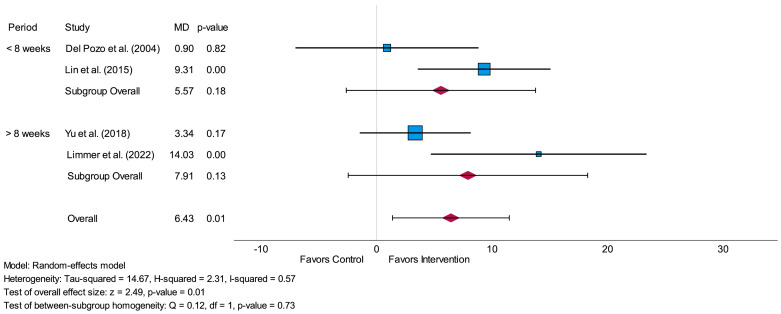
Forest plot of subgroup-analysis related to the time-domain parameter SDNN measured in milliseconds (short-term) comparing the impact of an intervention period of less than 8 weeks to an intervention period of more than 8 weeks. MD = mean difference [29,30,31,32].

Subgroup-analysis of SDNN for short-term assessment showed no significant difference (*p* = 0.73) between the MDs concerning an intervention period less than 8 weeks and an intervention period more than 8 weeks. There were no significant values either for an intervention period of less than 8 weeks (*p* = 0.18) or for an intervention period of more than 8 weeks (*p* = 0.13). The forest plot of this subgroup-analysis is presented in Figure 4.

**Figure 5:**
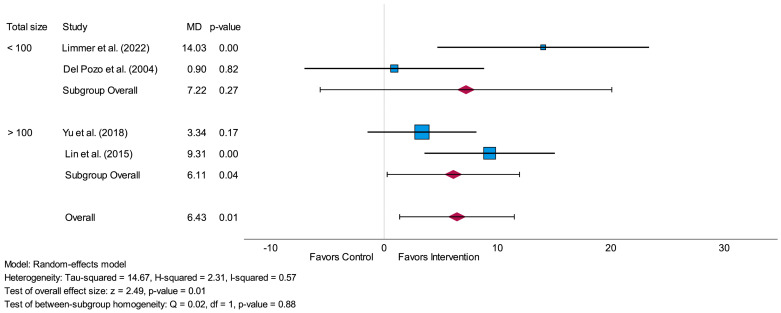
Forest plot of subgroup-analysis related to the time-domain parameter SDNN measured in milliseconds (short-term) comparing the results of studies with a total sample size of fewer than 100 participants to studies with a total sample size of more than 100 participants. MD = mean difference [29,30,31,32].

The subgroup-analysis related to the total amount of analyzed participants led to a non-significant difference (*p* = 0.88) between studies with fewer than 100 participants and studies with more than 100 participants. The overall effect size of the group with fewer than 100 participants was higher (MD = 7.22) in comparison to the overall effect size of the group with more than 100 participants (MD = 6.11). A significant result was only shown for the subgroup including more than 100 participants (*p* = 0.04 vs. *p* = 0.27). The forest plot related to this subgroup-analysis is shown in Figure 5.

#### 3.4.3. Total Power (TP)

The meta-analysis of TP (short-term) was conducted with two studies and included 119 participants in the intervention group and 107 participants in the control group [29,37]. One study investigated patients with CAD [29] whereas the other study focused on patients with CHF [37]. The overall effect size between the intervention group and the control group led to a high value (MD = 86.70) with no observed heterogeneity (I^2^ = 0.00) and no significance (*p* = 0.11). The resulting forest plot is presented in Figure S3 of the Supplementary Materials. 

#### 3.4.4. Low-Frequency Power (LF)

A total of 135 participants in the intervention group and 126 participants in the control group from two included studies were analyzed within the meta-analysis of the parameter LF (short-term) [29,32]. The meta-analysis resulted in an overall high effect size (MD = 160.11), which led to a significant result (*p* < 0.001), as shown in the forest plot in Figure S4 of the Supplementary Materials. Heterogeneity was not present (I^2^ = 0.00) within this analysis. In both studies, patients with CAD were investigated, and HRV-biofeedback was used as an intervention [29,32].

#### 3.4.5. High-Frequency Power (HF) and HF in Normalized Units (nHF)

Within the meta-analysis of HF (short-term), 135 participants were analyzed in the intervention group and 126 participants in the control group from two included studies [29,32]. Like the previous meta-analysis for LF, both studies investigated CAD patients and analyzed the impact of HRV-biofeedback as an intervention [29,32]. The analysis resulted in a small overall effect size (MD = 5.17) with no significance (*p* = 0.90) and a considerable heterogeneity (I^2^ = 0.78). The forest plot of this analysis is presented in Figure S5 of the Supplementary Materials.

The meta-analysis of nHF (short-term) was conducted with two studies and included 56 participants in the intervention and 61 participants in the control group [37,40]. One study investigated participants after stroke [40], while the other study investigated CHF patients [37]. The meta-analysis resulted in a medium overall effect size (MD = 9.74) with considerable heterogeneity (I^2^ = 0.91) and no significance (*p* = 0.41). The resulting forest plot is shown in Figure S6 of the Supplementary Materials.

### 3.5. Stress Management Interventions

#### 3.5.1. HRV-Biofeedback

Four studies investigated HRV-biofeedback as an intervention for stress management and were deemed eligible for meta-analysis [29,30,31,32]. The analyzed outcome parameters linked to HRV-biofeedback were SDNN, TP, LF and HF. The effect sizes for SDNN (short-term) led to small (MD = 0.90) up to high values (MD = 14.03). None of the analyzed studies showed lower SDNN (short-term) values in the intervention group in comparison to the control group after the intervention period [29,30,31,32]. For the meta-analysis of SDNN (24 h) the only study investigating HRV-biofeedback as an intervention resulted in no difference (MD = 0.00) between the intervention group and the control group after the completion of the intervention [30]. Concerning the analysis of the frequency-domain parameter TP, the analyzed study resulted in a high effect size (MD = 94.29) and showed higher post-interventional values in the intervention group in comparison to the control group [29]. Regarding the frequency-domain parameters LF and HF, one study reported higher mean values for both parameters in the intervention group compared to the control group after the intervention period [32]. The effect sizes led to a very high value (MD = 163.91) for LF and a high value (MD = 56.38) for HF. The other study reported higher post-intervention values in the intervention group for the parameter LF and lower post-interventional values in the intervention group compared to the control group for the parameter HF [29]. The effect size led to a high value for LF (MD = 156.35) and to a medium value for HF (MD = −28.91).

#### 3.5.2. Tai Chi

Tai chi as intervention for stress management was investigated in two studies that were included in meta-analysis [35,40]. Analyzed outcome parameters associated with tai chi as a stress reduction intervention were derived from a meta-analysis for the outcome parameters CRP and nHF. Regarding the meta-analysis of CRP, the analyzed study investigating tai chi as intervention reported a higher CRP level in the intervention group in comparison to the control group after the intervention period [35]. The effect size was high (MD = 2.30), favoring the control group. Concerning the analysis of the frequency-domain parameter nHF, the analyzed study showed lower post-interventional values in the intervention group compared to the control group [40]. The effect size resulted in a small value (MD = −2.90) favoring the control group.

#### 3.5.3. Yoga

Three studies examining yoga as an intervention were all deemed suitable for meta-analysis [34,37,41,42]. Findings concerning the effectiveness of yoga for stress reduction were compiled from a meta-analysis focusing on outcome measures such as CRP, SDNN, TP and nHF. For meta-analysis of CRP, the analyzed study reported lower post-interventional values in the intervention group compared to the control group [34]. The effect size led to a small value (MD = −0.72), favoring the intervention group. For the parameter SDNN (24 h), higher mean values were reported for the intervention group in comparison to the control group after the intervention period [41,42]. This led to a high effect size (MD = 12.00). Similar results were investigated for the frequency-domain parameters TP and nHF. The analyzed study investigating yoga as an intervention reported higher post-interventional values in the intervention group compared to the control group for both parameters [37]. For the parameter TP, the effect size led to a small value (MD = 8.33), and for nHF, it led to a high value (MD = 20.63). 

### 3.6. Sensitivity Analysis

Similar to the analysis of the effect size MD, sensitivity analysis using the effect size SMD showed a significant result for the time-domain parameter SDNN regarding short-term assessment (*p* = 0.01) and the frequency-domain parameter LF (*p* < 0.001). Within the sensitivity analysis, the time-domain parameter SDNN for 24 h assessment was not significant (*p* = 0.36) compared to the conducted analysis with the effect size MD. A high effect size was estimated for the frequency-domain parameters LF (SMD = 0.81) and nHF (SMD = 1.08), a medium effect size for the time-domain parameter SDNN regarding 24 h assessment (SMD = 0.61), a low effect size for the time-domain parameter SDNN related to short-term assessment (SMD = 0.41) and a very low effect size for CRP (SMD = 0.06), the frequency-domain parameter TP (SMD = 0.17) as well as HF (SMD = −0.15). Table S4 of the Supplementary Materials summarizes the effect size with its confidence interval, the standard error and *p*-value for each analyzed parameter.

## 4. Discussion

As part of a systematic review and meta-analysis, the authors evaluated the impact of stress-reducing interventions such as HRV-biofeedback, tai chi and yoga on the biomarker CRP and the time-domain and frequency-domain parameters of HRV. Significant changes between the intervention and control groups were identified for the time-domain parameters SDNN (short-term) and SDNN (24 h), as well as for the frequency-domain parameter LF (short-term). Notably, the analysis of SDNN (short-term) and LF (short-term) only included studies with HRV-biofeedback as an intervention [29,30,31,32]. This suggests that HRV-biofeedback has a beneficial effect in cardiovascular patients by augmenting the HRV. 

The significance of increased HRV should not be underestimated, as reduced HRV is associated with various forms of CVD, such as ischemia or cardiac arrhythmia [57,58,59,60]. Reduced HRV has been also linked to an increased risk of mortality, particularly in patients with CAD or CHF [61]. Risk factors like diabetes, hyperglycemia and insulin resistance were shown to impact the adaptability of the cardiovascular system and decrease HRV [60]. Mental stress is also strongly linked to reduced HRV, with stress being recognized as a significant contributor to the development and progression of CVD [5,60,62]. These findings underscore the importance of implementing stress reduction techniques in cardiac rehabilitation programs and integrating them into the daily lives of patients with CVD. Doing so could significantly reduce the risk of future events and improve overall cardiovascular health outcomes.

HRV-biofeedback has been identified as an effective method for reducing stress, according to a previously published meta-analysis indicating lower self-reported stress levels and highlighting the benefits of HRV-biofeedback as a stress reduction intervention [63]. The efficacy of HRV-biofeedback in reducing stress was also demonstrated in a prior trial emphasizing that HRV-biofeedback is a readily accessible approach to stress reduction, comparable to physical activity and mindfulness-based meditation [64]. These findings are consistent with results presented in the current meta-analysis, which underline the benefits of increasing HRV through HRV-biofeedback. Overall, the collective evidence from this meta-analysis and previous literature underscores the effectiveness and accessibility of HRV-biofeedback as a valuable tool to increase HRV with potential implications for improving overall well-being and cardiovascular health.

Mind–body exercises such as tai chi and yoga demonstrate notable efficacy in reducing stress, according to a previously conducted meta-analysis [65]. Our own meta-analysis further corroborates the benefits of yoga. In our study, yoga yielded significant improvement in the SDNN (24 h) parameter with a notably high effect size compared to HRV-biofeedback intervention [30,41,42]. Additionally, both of our meta-analyses highlight the positive impact of mind–body exercises on nHF values [65]. While yoga consistently showed favorable outcomes in increasing nHF, the results for tai chi were not as pronounced. Our meta-analysis examining CRP levels revealed that yoga led to lower CRP levels in the intervention group compared to the control group [34]. This is particularly relevant, considering the association between mental stress and increased inflammatory markers in patients with CAD [66]. Yoga emerges as an effective stress reduction method, not only by enhancing HRV but also by reducing CRP levels in patients with CVD [34]. The inclusion of meditation sessions in yoga practice sessions further augments yoga’s stress-reducing effects. Prior research has consistently demonstrated the efficacy of mindfulness-based meditation in alleviating stress, reinforcing the holistic benefits of mind–body exercises such as yoga, which the authors discussed here [64].

Recently, a meta-analysis was published which, similar to our meta-analysis, also focused on stress-reducing interventions and their effect on HRV. Since this meta-analysis also analyzed some studies that were examined in our own work, our findings arrived at partially consistent conclusions [67]. This also implies that our meta-analysis is not the first to investigate the effect of stress-reducing interventions on HRV in patients with CVD. However, our meta-analysis differs in that we also considered other parameters of HRV (TP, LF, and nHF), as well as biomarkers such as CRP, for investigation, aiming to examine the influence of stress-reducing interventions on multiple parameters.

The sensitivity analysis conducted using the effect size SMD revealed significant results for the time-domain parameter SDNN (short-term) and the frequency-domain parameter LF (short-term). The time-domain parameter SDNN (24 h) did not yield significant results compared to the analysis utilizing MD as an effect size. Nevertheless, the sensitivity analysis highlighted differences between the intervention and control groups for all parameters examined.

Our meta-analysis has several limitations. Firstly, an in-depth analysis of the causes of heterogeneity was not feasible due to the inclusion of the small number of studies in all conducted meta-analyses. Secondly, the sample sizes in our meta-analyses were relatively small, which may affect the robustness and generalizability of our findings. Furthermore, studies were classified based on the main CVD examined in each study. It is worth noting that this classification does not necessarily exclude the possibility that some participants might have had other cardiovascular diagnoses unrelated to those conditions which were the focus of the study. In one of the analyzed studies, the pre-interventional CRP values of the control group were notably lower than those of the intervention group [35]. This discrepancy in baseline values could potentially impact the resulting effect sizes. In cases where the pre-intervention values of the control group were significantly higher than those of the intervention group, the effect sizes related to HRV parameters might also be impacted. These limitations underscore the need for cautious interpretation of our findings and highlight areas for future research with larger sample sizes and more comprehensive methodologies.

In summary, this meta-analysis suggests that stress-reducing interventions, particularly HRV-biofeedback and yoga, offer significant benefits in enhancing HRV among patients with CVD. Given these findings, it is imperative to consider implementing stress reduction interventions not only for patients with established CVD but also for those at risk of future development and progression of heart disease.

## Figures and Tables

**Table 1:** Characteristics of the 12 included studies.

Author	Year	Main Disease	Intervention	Intervention Period	Outcome of Interest	Sample Size		Mean Age	Female
Claesson et al. [33]	2006	Ischemic heart disease	Cognitive behavioral stress management	1 year	Fibrinogen, hsCRP	Intervention Control	77 82	59.4 ± 9.3 62.2 ± 7.7	77 82
Jain et al. [34]	2022	Congestive heart failure	Yoga	3 months	C-reactive protein	Intervention Control	30 30	51.9 ± 6.9 52.3 ± 6.6	6 9
Redwine et al. [35]	2020	Congestive heart failure	Tai chi	16 weeks	C-reactive protein	Intervention Control	24 * 23 *	63.0 ± 9.0 67.0 ± 7.0	2 3
Yu et al. [29]	2018	Coronary artery disease	HRV-biofeedback	18 weeks	Breathing rate, heart rate variability	Intervention Control	75 59	61.2 ± 7. 4 60. 3 ± 6.9	9 6
Limmer et al. [30]	2022	Myocardial infarction	HRV-biofeedback	12 weeks	Breathing rate, heart rate variability	Intervention Control	23 * 23 *	57.4 ± 8.8 63.6 ± 9.9	2 5
Chevalier et al. [36]	2004	Ventricular tachyarrhythmias	Cognitive behavioral therapy	3 months	Heart rate variability	Intervention Control	35 * 35 *	58.5 ± 10.0 57.9 ± 11.0	5 1
Del Pozo et al. [31]	2004	Coronary artery disease	HRV-biofeedback	6 weeks	Heart rate variability	Intervention Control	31 32	66.8 ± 8.4 68.0 ± 9.0	11 10
Krishna et al. [37,38]	2014	Congestive heart failure	Yoga	12 weeks	Heart rate variability, hsCRP	Intervention Control	44 48	49.3 ± 5.7 50.1 ± 4.5	12 16
Blumenthal et al. [39]	2005	Ischemic heart disease	Cognitive social learning model of behavior	16 weeks	Heart rate variability	Intervention Control	44 * 42 *	63.0 ± 11.5 63.0 ± 9.0	15 10
Chan and Tsang [40]	2020	Stroke	Tai chi	12 weeks	Heart rate variability	Intervention Control	19 * 19 *	64.2 ± 8.2 61.8 ± 7.3	9 11
Lin et al. [32]	2015	Coronary artery disease	HRV-biofeedback	6 weeks	Heart rate variability	Intervention Control	77 * 77 *	61.0 ± 8.4 60.6 ± 8.0	6 11
Lopes et al. [41,42]	2017	Congestive heart failure	Yoga	8 weeks	Heart rate variability	Intervention Control	11 10	67.0 ± 6.0 62.0 ± 6.0	10 6

hsCRP = high-sensitivity c-reactive protein; * Number of analyzed participants for at least one outcome parameter of interest different from the mentioned sample size.

## Data Availability

No new data were created or analyzed in this study. Data sharing is not applicable to this article.

## Supplementary Materials

The following are available online at https://www.mdpi.com/article/10.3390/life14060749/s1, Table S1. Used search terms and the number of results. Table S2. Characteristics of eligible ongoing randomized controlled trials. Table S3. Characteristics of completed randomized controlled trials without access to the results or without published results. Table S4. Effect sizes, confidence intervals, standard errors and *p*-values resulting from sensitivity analysis using standard mean difference as effect size. Figure S1. Forest plot showing the mean differences of c-reactive protein levels measured in milligrams per liter between the intervention group and the control group in patients with congestive heart failure. SD = standard deviation, MD = mean difference, CI = confidence interval. Figure S2. Forest plot presenting the mean differences of SDNN measured in milliseconds (24-h) between the intervention group and the control group. SDNN = standard deviation of Normal-to-Normal intervals, SD = standard deviation, MD = mean difference, CI = confidence interval. Figure S3. Forest plot illustrating the mean differences of TP measured in square milliseconds (short-term) between the intervention group and the control group. TP = total power, SD = standard deviation, MD = mean difference, CI = confidence interval. Figure S4. Forest plot showing the mean differences of LF measured in square milliseconds (short-term) between the intervention group and the control group. LF = low-frequency power, SD = standard deviation, MD = mean difference, CI = confidence interval. Figure S5. Forest plot illustrating the mean differences of HF measured in square milliseconds (short-term) between the intervention group and the control group. HF = high-frequency power, SD = standard deviation, MD = mean difference, CI = confidence interval. Figure S6. Forest plot showing the mean differences of nHF (short-term) between the intervention group and the control group. nHF = high-frequency power in normalized units, SD = standard deviation, MD = mean difference, CI = confidence interval.

## Abbreviations

CAD	coronary artery disease
CHF	congestive heart failure
CRP	c-reactive protein
CVD	cardiovascular disease
ECG	electrocardiogram
HF	high-frequency power
HRV	heart rate variability
hsCRP	high-sensitivity c-reactive protein
LF	low-frequency power
MD	mean difference
MeSH	Medical Subject Headings
MI	myocardial infarction
nHF	HF in normalized units
nLF	LF in normalized units
PRISMA	Preferred Reporting of Items for Systematic Meta-analysis
PROSPERO	International Prospective Register of Systematic Reviews
PSNS	parasympathetic nervous system
RCT	randomized controlled trial
SD	standard deviation
SDNN	standard deviation of Normal-to-Normal intervals
SMD	standard mean difference
SNS	sympathetic nervous system
TP	total power
