# Supplementary material for: The Effect of Stress-Reducing Interventions on Heart Rate Variability in Cardiovascular Disease: A Systematic Review and Meta-Analysis
Source: Life (Basel). 2024 Jun 12;14(6):749. doi: 10.3390/life14060749 (PMC11204824; doi:10.3390/life14060749)
Supplement: Supplementary file 1 [file life-14-00749-s001.zip › life-3019082-supplementary.pdf]

Supplemental Material

The Effect of Stress-Reducing Interventions on Heart Rate Variability in Cardiovascular Disease: A Systematic Review and Meta-Analysis

Ouahiba El-Malahi [1], Darya Mohajeri [1], Alexander Bäuerle [2,3], Raluca Mincu [1], Korbinian Rothenaicher [1], Greta Ullrich [1], Christos Rammos [1], Martin Teufel [2,3], Tienush Rassaf [1] and Julia Lortz [1,*]

[1] Department of Cardiology and Vascular Medicine, West-German Heart and Vascular Center Essen, University of Duisburg-Essen, Hufelandstr. 55, 45147 Essen, Germany
[2] Clinic for Psychosomatic Medicine and Psychotherapy, LVR-University Hospital Essen, University of Duisburg-Essen, Virchowstr. 174, 45147 Essen, Germany
[3] Center for Translational Neuro- and Behavioral Sciences (C-TNBS), University of Duisburg-Essen, 45147 Essen, Germany

* Correspondence: julia.lortz@uk-essen.de; Tel.: +49-201-723-84995

Index

Method ... 2

Search strategy ... 2

Results ... 8

Study selection and characteristics ... 8

Meta-analysis ... 11

C-reactive protein (CRP) ... 11

Standard deviation of Normal-to-Normal intervals (SDNN) ... 11

Total power (TP) ... 12

Low-frequency power (LF) ... 12

High-frequency power (HF) ... 12

High-frequency power in normalized units (nHF) ... 13

Sensitivity analysis ... 13

References ... 14

Method

Search strategy

Table S1. Used search terms and the number of results

Search	Source and date	Search term	Number of results
**1**	Pubmed 03.05.2023	("major adverse cardiovascular event" OR "myocardial infarction" OR "coronary infarction" OR "cardiac infarction" OR "ischemic heart disease" OR "heart failure" OR stroke OR "peripheral occlusive disease" OR "coronary revascularization" OR "cardiac arrhythmia" OR "cardiovascular disease" OR "coronary artery disease" OR "acute coronary syndrome" OR "cardiac arrest" OR "heart arrest" OR "heart attack" OR "atrial fibrillation" OR "Cardiovascular Diseases"[Mesh] OR "Stroke"[Mesh] OR "Percutaneous Coronary Intervention"[Mesh] OR "Arrhythmias, Cardiac"[Mesh] OR "Atrial Fibrillation"[Mesh]) AND ("stress management" OR "stress reduction" OR "yoga" OR "biofeedback" OR "behavioral therapy" OR "acupuncture" OR "mind-body" OR "mindfulness-based" OR "meditation" OR "Tai Chi" OR "Yoga"[Mesh] OR "Biofeedback, Psychology"[Mesh] OR "Cognitive Behavioral Therapy"[Mesh] OR "Acupuncture Therapy"[Mesh] OR "Mind-Body Therapies"[Mesh] OR "Tai Ji"[Mesh]) AND ("heart rate variability" OR "c-reactive protein" OR "cortisol" OR "fibrinogen" OR "respiratory rate" OR "blink rate" OR "C-Reactive Protein"[Mesh] OR "Hydrocortisone"[Mesh] OR "Fibrinogen"[Mesh] OR "Respiratory Rate"[Mesh] OR "Blinking"[Mesh]) AND ("randomized controlled trial" OR "randomized controlled study" OR random*)	206

**2**	Embase 03.05.2023	('major adverse cardiovascular event' OR 'myocardial infarction' OR 'coronary infarction' OR 'cardiac infarction' OR 'ischemic heart disease' OR 'heart failure' OR stroke OR 'peripheral occlusive disease' OR 'coronary revascularization' OR 'cardiac arrhythmia' OR 'cardiovascular disease' OR 'coronary artery disease' OR 'acute coronary syndrome' OR 'cardiac arrest' OR 'heart arrest' OR 'heart attack' OR 'atrial fibrillation' OR 'Cardiovascular Disease'/exp OR 'cerebrovascular accident'/exp OR 'Percutaneous Coronary Intervention'/exp) AND ('stress management' OR 'stress reduction' OR yoga OR biofeedback OR 'behavioral therapy' OR acupuncture OR mind-body OR mindfulness-based OR meditation OR 'Tai Chi' OR Yoga/exp OR 'Biofeedback'/exp OR 'Cognitive Behavioral Therapy'/exp OR 'Acupuncture'/exp OR 'Alternative Medicine'/exp OR 'Tai Chi'/exp) AND ('heart rate variability' OR 'c-reactive protein' OR cortisol OR fibrinogen OR 'respiratory rate' OR 'blink rate' OR 'C Reactive Protein'/exp OR Hydrocortisone/exp OR Fibrinogen/exp OR 'Breathing Rate'/exp OR Blinking/exp) AND ('randomized controlled trial' OR 'randomized controlled study' OR random*) *(converted by using Polyglot [1] and terms from Emtree are already included)*	488
**3**	Cochrane Library 03.05.2023	("major adverse cardiovascular event" OR "myocardial infarction" OR "coronary infarction" OR "cardiac infarction" OR "ischemic heart disease" OR "heart failure" OR stroke OR "peripheral occlusive disease" OR "coronary revascularization" OR "cardiac arrhythmia" OR "cardiovascular disease" OR "coronary artery disease" OR "acute coronary	269

		syndrome" OR "cardiac arrest" OR "heart arrest" OR "heart attack" OR "atrial fibrillation") AND ("stress management" OR "stress reduction" OR yoga OR biofeedback OR "behavioral therapy" OR acupuncture OR mind-body OR mindfulness-based OR meditation OR "Tai Chi") AND ("heart rate variability" OR "c-reactive protein" OR cortisol OR fibrinogen OR "respiratory rate" OR "blink rate")<br>*(converted by using Polyglot [1] and MeSH terms are not included)*	
**4**	ClinicalTrials.gov<br>03.05.2023	*Search field "Condition or disease":*<br>("major adverse cardiovascular event" OR "myocardial infarction" OR "ischemic heart disease" OR "heart failure" OR stroke OR "peripheral occlusive disease" OR "coronary revascularization" OR "heart attack" OR "atrial fibrillation")<br>*Search field "Other terms":*<br>("stress management" OR yoga OR biofeedback OR "behavioral therapy" OR acupuncture OR "mind-body" OR "mindfulness-based" OR meditation OR "Tai Chi") AND ("heart rate variability" OR "c-reactive protein" OR cortisol OR fibrinogen)	50
**5**		*Search field "Condition or disease":*<br>("major adverse cardiovascular event" OR "myocardial infarction" OR "ischemic heart disease" OR "heart failure" OR stroke OR "peripheral occlusive disease" OR "coronary revascularization" OR "heart attack" OR "atrial fibrillation")<br>*Search field "Other terms":*<br>("stress management" OR yoga OR biofeedback OR "behavioral therapy" OR acupuncture OR "mind-body" OR "mindfulness-based" OR	22

		meditation OR "Tai Chi") AND ("respiratory rate" OR "blink rate")	
**6**		*Search field "Condition or disease":*<br>("coronary infarction" OR "cardiac infarction" OR "cardiac arrhythmia" OR "cardiovascular disease" OR "coronary artery disease" OR "acute coronary syndrome" OR "cardiac arrest" OR "heart arrest")<br>*Search field "Other terms":*<br>("stress management" OR yoga OR biofeedback OR "behavioral therapy" OR acupuncture OR "mind-body" OR "mindfulness-based" OR meditation OR "Tai Chi") AND ("heart rate variability" OR "c-reactive protein" OR cortisol OR fibrinogen)	97
**7**		*Search field "Condition or disease":*<br>("coronary infarction" OR "cardiac infarction" OR "cardiac arrhythmia" OR "cardiovascular disease" OR "coronary artery disease" OR "acute coronary syndrome" OR "cardiac arrest" OR "heart arrest")<br>*Search field "Other terms":*<br>("stress management" OR yoga OR biofeedback OR "behavioral therapy" OR acupuncture OR "mind-body" OR "mindfulness-based" OR meditation OR "Tai Chi") AND ("respiratory rate" OR "blink rate")	49
**8**	German Clinical Trials Register (GermanCTR) 03.05.2023	('major adverse cardiovascular event' OR 'myocardial infarction' OR 'coronary infarction' OR 'cardiac infarction' OR 'ischemic heart disease' OR 'heart failure' OR stroke OR 'peripheral occlusive disease' OR 'coronary revascularization' OR 'cardiac arrhythmia' OR 'cardiovascular disease' OR 'coronary artery disease' OR 'acute coronary syndrome' OR 'cardiac arrest' OR 'heart arrest' OR 'heart attack' OR 'atrial fibrillation') AND ('stress	0

		management' OR 'stress reduction' OR yoga OR biofeedback OR 'behavioral therapy' OR acupuncture OR mind-body OR mindfulness-based OR meditation OR 'Tai Chi') AND ('heart rate variability' OR 'c-reactive protein' OR cortisol OR fibrinogen OR 'respiratory rate' OR 'blink rate')	
**9**	ICTRP 03.05.2023	("major adverse cardiovascular event" OR "myocardial infarction" OR “coronary infarction” OR “cardiac infarction” OR "ischemic heart disease" OR "heart failure" OR stroke OR "peripheral occlusive disease" OR "coronary revascularization" OR "cardiac arrhythmia" OR "cardiovascular disease" OR “coronary artery disease” OR "acute coronary syndrome" OR "cardiac arrest" OR “heart arrest” OR “heart attack” OR "atrial fibrillation”) AND ("stress management" OR "stress reduction" OR "yoga” OR "biofeedback” OR "behavioral therapy" OR "acupuncture” OR "mind-body" OR "mindfulness-based" OR “meditation” OR “Tai Chi”) AND ("heart rate variability" OR "c-reactive protein" OR "cortisol” OR “fibrinogen” OR "respiratory rate" OR “blink rate“)	12
**10**	ISRCTN registry 03.05.2023	("major adverse cardiovascular event" OR "myocardial infarction" OR “coronary infarction” OR “cardiac infarction” OR "ischemic heart disease" OR "heart failure" OR stroke OR "peripheral occlusive disease" OR "coronary revascularization" OR "cardiac arrhythmia" OR "cardiovascular disease" OR “coronary artery disease” OR "acute coronary syndrome" OR "cardiac arrest" OR “heart arrest” OR “heart attack” OR "atrial fibrillation”) AND ("stress management" OR "stress reduction" OR "yoga” OR "biofeedback”	8

		OR "behavioral therapy" OR "acupuncture" OR "mind-body" OR "mindfulness-based" OR "meditation" OR "Tai Chi") AND ("heart rate variability" OR "c-reactive protein" OR "cortisol" OR "fibrinogen" OR "respiratory rate" OR "blink rate")	
**Total**			**1201**
**Total after removing duplicates**			**780**

Results

Study selection and characteristics

Table S2. Characteristics of eligible ongoing randomized controlled trials

Study name	Participants	Interventions	Outcome of interest	Starting date	Notes
"Online Cognitive Behavioral Therapy Targeting Cardiac Anxiety Following Myocardial Infarction: A Randomized Controlled Trial" [2] (NCT05580718)	Adults (18-80 years old) with a previous myocardial infarction and cardiac anxiety	Intervention: "Internet-delivered exposure-based cognitive behavioral therapy" [2]<br><br>Control (waitlist): Usual care	Cortisol, high-sensitivity c-reactive protein	October 2022	Outcome of interest: Number of patients with high laboratory values is mentioned as outcome, however values related to these parameters could be posted as well.
"Screening and Intervention Reducing Anxiety in Patients With Cardiac Disease: The Heart & Mind Trial" [3, 4] (NCT04582734)	Adults (≥ 18 years old) with cardiac disease and symptoms of anxiety (HADS-A score ≥ 8, a greater HADS-A score than the HADS-D score)	Intervention: Cognitive behavioral therapy in addition to usual care<br><br>Control: Usual care	Cortisol, c-reactive protein, heart rate variability	June 2021	Clinical trial protocol published with further details to the study.
"The Effect of Mindfulness on Vascular Inflammation in Stable Coronary Disease: A Multi-System PET/MRI Study" [5] (NCT04505865)	Adults (50-65 years old) with cardiovascular disease and stress (PSS score over 13 and/or feeling stressed)	Intervention: Mindfulness-based stress reduction in addition to usual care<br><br>Control: Usual care	High-sensitivity c-reactive protein	November 2021	None
"Effectiveness of Mindfulness-based Stress Reduction for Improving Quality of Life in Patients With Cardiovascular Disease: a Randomised Controlled Trial" [6] (NCT03826836)	Adults (≥ 18 years old) with atherosclerotic cardiovascular disease	Intervention: Mindfulness-based stress reduction in addition to usual care<br><br>Control: Usual care	Cortisol	January 2019	None

Study name	Participants	Interventions	Outcome of interest	Starting date	Notes
"Study on the effect of acupunture treatment on autonomic nerve dysfunction in convalescent period of stroke based on heart rate variability assessment technique" [7, 8] (ChiCTR2100052417)	Adults (18-90 years old) with cerebrovascular disease	Intervention:<br>Acupuncture in addition to drugs for symptomatic treatment<br><br>Control: Drugs for symptomatic treatment	Heart rate variability	November 2021	Clinical trial protocol published with further details to the study.

Table S3. Characteristics of completed randomized controlled trials without access to the results or without published results

Study name	Participants	Interventions	Outcome of interest	Starting date	Notes
"Development and Evaluation of a Web-based Group Cognitive-behavioral Therapy Program for Coronary Artery Heart Disease Patients" [9] (NCT01998555)	Adults (30-70 years old) with coronary artery disease	Intervention: Cognitive behavioral therapy<br><br>Control: Waitlist	Breathing rate	December 2013	No results found. Outcome parameter is not described in detail. E-mail address of the named contact person was not found. Another person related to this study was contacted - no response received.
"Investigating the Impact of Mindfulness on the Physiological and Psychological Well-being of Stroke Survivors and Their Caregivers" [10] (NCT03659409)	Adults (21-80 years old) who survived a stroke and their familiy caregivers	Intervention: Mindfulness-based program<br><br>Control: Waitlist	Heart rate variability	May 2016	No results found. An e-mail was sent to the named contact person. Answer received, however contact person is not able to send the results.
"Randomised controlled trial of a 12 week yoga intervention on negative affective states, cardiovascular and cognitive function in post-cardiac rehabilitation patients" [11, 12] (ACTRN12612000358842)	Adults (>18 years old) with coronary artery disease, completed phase 2 cardiac rehabilitation program (≤ 9 months)	Intervention: Yoga<br><br>Control: Waitlist	Heart rate variability, c-reactive protein	April 2012	Clinical trial protocol was found via a Google search and provides further information. No results found for this study and no response received after contacting the first author.
"The treatment to the autonomic dysfunction in acute stroke by auricular acupuncture mediated by ABVN:a clinical study" [13] (ChiCTR-IPR-17011893)	Cerebrovascular disease, admission within 3 days after initial stroke and hospitalization with stable condition	Intervention: Acupuncture<br><br>Control: Usual care	Heart rate variability	August 2017	No results found. Request was sent to the contact person, however the e-mail returned with an error.

Meta-analysis[1]

9 studies were considered eligible for meta-analysis [14-23].

C-reactive protein (CRP)

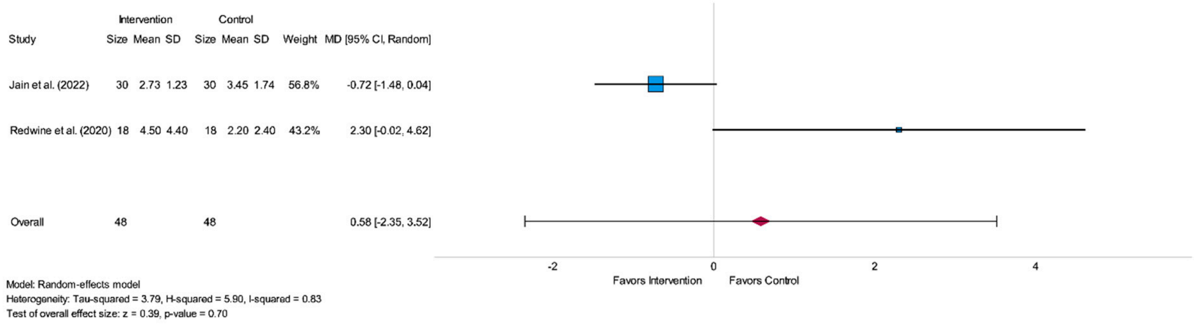

Figure S1. Forest plot showing the mean differences of c-reactive protein levels measured in milligrams per liter between the intervention group and the control group in patients with congestive heart failure. SD = standard deviation, MD = mean difference, CI = confidence interval

Standard deviation of Normal-to-Normal intervals (SDNN)

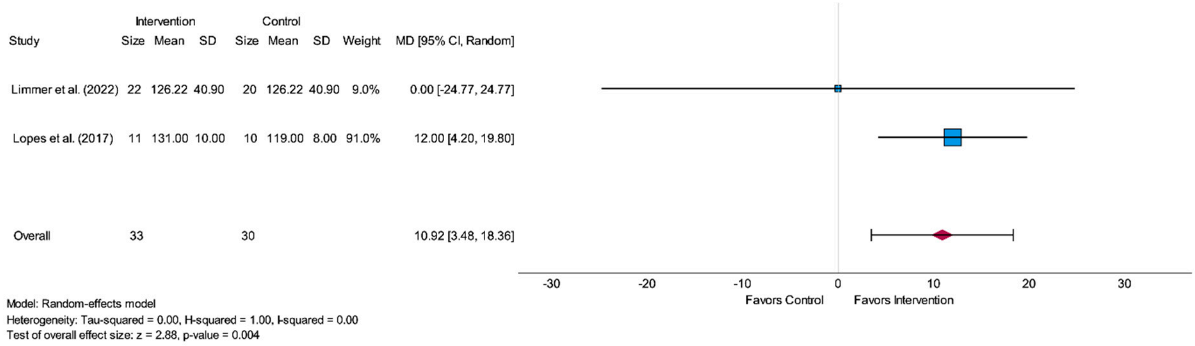

Figure S2. Forest plot presenting the mean differences of SDNN measured in milliseconds (24-hour) between the intervention group and the control group. SDNN = standard deviation of Normal-to-Normal intervals, SD = standard deviation, MD = mean difference, CI = confidence interval

[1] The forest plots can be increased by using the zoom function.

Total power (TP)

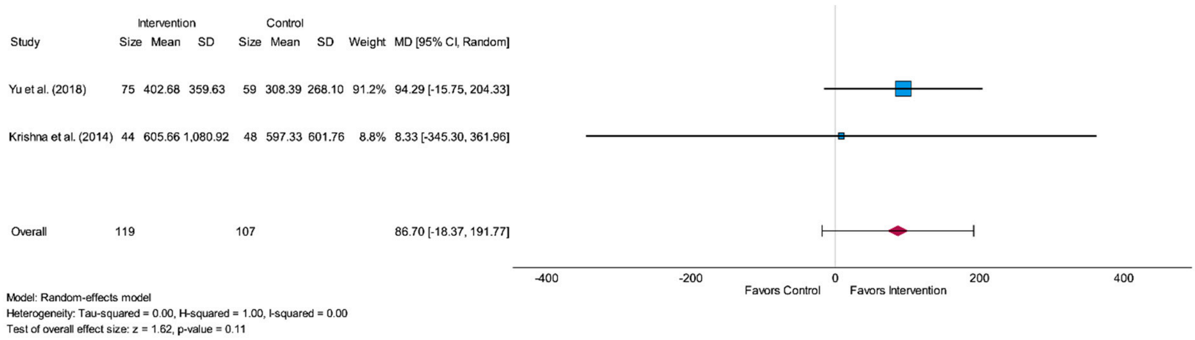

Figure S3. Forest plot illustrating the mean differences of TP measured in square milliseconds (short-term) between the intervention group and the control group. TP = total power, SD = standard deviation, MD = mean difference, CI = confidence interval

Low-frequency power (LF)

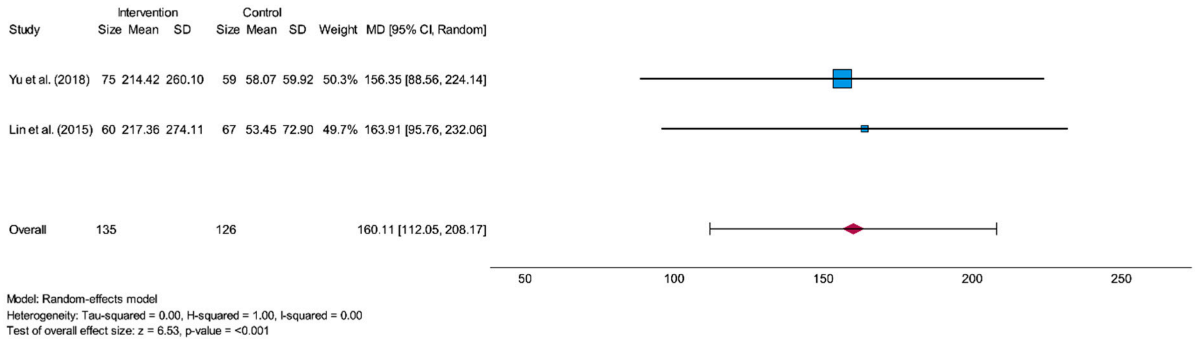

Figure S4. Forest plot showing the mean differences of LF measured in square milliseconds (short-term) between the intervention group and the control group. LF = low-frequency power, SD = standard deviation, MD = mean difference, CI = confidence interval

High-frequency power (HF)

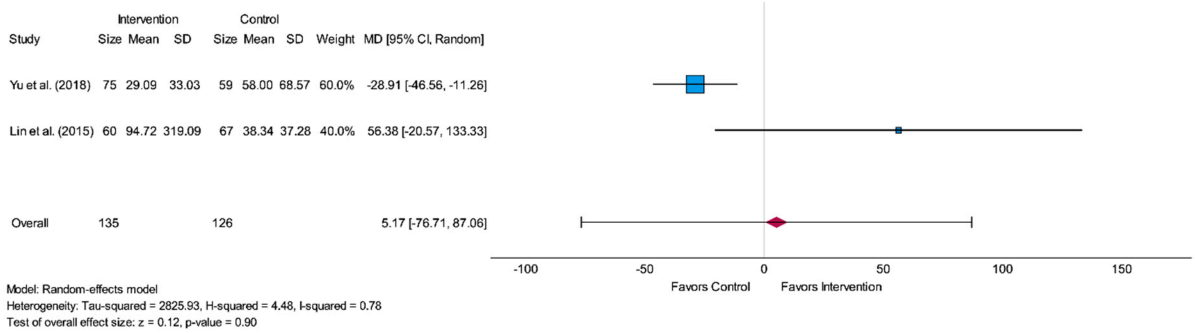

Figure S5. Forest plot illustrating the mean differences of HF measured in square milliseconds (short-term) between the intervention group and the control group. HF = high-frequency power, SD = standard deviation, MD = mean difference, CI = confidence interval

High-frequency power in normalized units (nHF)

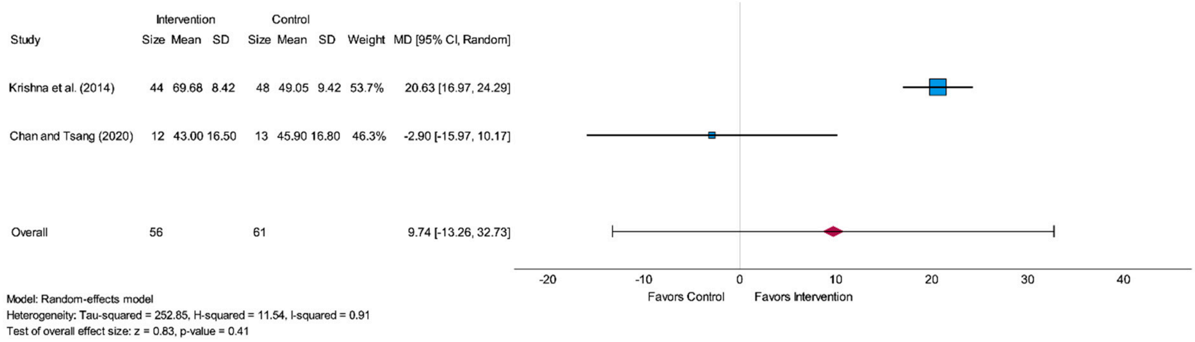

Figure S6. Forest plot showing the mean differences of nHF (short-term) between the intervention group and the control group. nHF = high-frequency power in normalized units, SD = standard deviation, MD = mean difference, CI = confidence interval

Sensitivity analysis

Table S4. Effect sizes, confidence intervals, standard errors and p-values resulting from sensitivity analysis using standard mean difference as effect size.

Outcome	Effect size [95 % CI]	Standard error	p-value
CRP	0.06 [-1.04, 1.17]	0.56	0.91
SDNN (short-term)	0.41 [0.10, 0.72]	0.16	0.01
SDNN (24-hour)	0.61 [-0.68, 1.89]	0.66	0.36
TP (short-term)	0.17 [-0.10, 0.45]	0.14	0.21
LF (short-term)	0.81 [0.56, 1.07]	0.13	<0.001
HF (short-term)	-0.15 [-0.95, 0.65]	0.41	0.71
nHF (short-term)	1.08 [-1.35, 3.51]	1.24	0.38

CRP = c-reactive protein, SDNN = standard deviation of Normal-to-Normal intervals, TP = total power, LF = low-frequency power, HF = high-frequency power, nHF = high-frequency power in normalized units

References

1. Clark, J.M.; Sanders, S.; Carter, M.; Honeyman, D.; Cleo, G.; Auld, Y.; Booth, D.; Condron, P.; Dalais, C.; Bateup, S.; et al. Im-proving the translation of search strategies using the Polyglot Search Translator: A randomized controlled trial. J. Med. Libr. Assoc. 2020, 108, 195–207. https://doi.org/10.5195/jmla.2020.834. PMID: 32256231.
2. NCT05580718. Online Cognitive Behavioral Therapy Targeting Cardiac Anxiety (MI-CBT) [Trial registry record]. Available online: https://ClinicalTrials.gov/show/NCT05580718 (accessed on 29 May 2023).
3. Berg, S.K.; Herning, M.; Schjødt, I.; Thorup, C.B.; Juul, C.; Svendsen, J.H.; Jorgensen, M.B.; Risom, S.S.; Christensen, S.W.; Thygesen, L.; et al. The heart & mind trial: Intervention with cognitive-behavioural therapy in patients with cardiac disease and anxiety: Randomised controlled trial protocol. BMJ Open 2021, 11, e057085. https://doi.org/10.1136/bmjopen-2021-057085. PMID: 34862302.
4. NCT04582734. Screening and Intervention Reducing Anxiety in Patients with Cardiac Disease [Trial registry record]. Available online: https://ClinicalTrials.gov/show/NCT04582734 (accessed on 29 May 2023).
5. NCT04505865. The Effect of Mindfulness on Vascular Inflammation in Stable Coronary Disease [Trial registry record]. Available online: https://ClinicalTrials.gov/show/NCT04505865 (accessed on 29 May 2023).
6. NCT03826836. Mind Our Heart Study [Trial Registry Record]. Available online: https://clinicaltrials.gov/show/NCT03826836 (accessed on 29 May 2023).
7. ChiCTR2100052417. Study on the Effect of Acupunture Treatment on Autonomic Nerve Dysfunction in Convalescent Period of Stroke Based on Heart Rate Variability Assessment Technique [Trial Registry Record]. Available online: https://trialsearch.who.int/Trial2.aspx?TrialID=ChiCTR2100052417 (accessed on 29 May 2023).
8. Jia S, Lu W, Hang M, Zhang C, Ma Z, Xue K, et al. Study on the effect of acupunture treatment on autonomic nerve dysfunction in convalescent period of stroke based on heart rate variability assessment technique. Medicine 2022;101:50(e32355). doi: 10.1097/md.0000000000032355. PMID: 36550889.
9. NCT01998555. Web-based Psychological Intervention to Coronary Artery Heart Disease Patients [Trial registry record]. Available online: https://ClinicalTrials.gov/show/NCT01998555 (accessed on 29 May 2023).
10. NCT03659409. Stroke Of Mindfulness: Investigating Physiological and Psychological Well-being [Trial registry record]. Available online: https://clinicaltrials.gov/show/NCT03659409 (accessed on 29 May 2023).
11. Yeung, A.; Kiat, H.; Denniss, A.R.; Cheema, B.S.; Bensoussan, A.; Machliss, B.; Colagiuri, B.; Chang, D. Randomised controlled trial of a 12 week yoga intervention on negative affective states, cardiovascular and cognitive function in post-cardiac rehabilitation patients. BMC Complement. Altern. Med. 2014, 14, 411. https://doi.org/10.1186/1472-6882-14-411. PMID: 25342209.
12. ACTRN12612000358842. Evaluation of a 12 week Yoga Intervention on Negative Affective States, Cardiovascular and Cognitive Function in Post-Cardiac Rehabilitation Patients [Trial registry record]. Available online: https://trialsearch.who.int/Trial2.aspx?TrialID=ACTRN12612000358842 (accessed on 29 May 2023).
13. ChiCTR-IPR-17011893. The Treatment to the Autonomic Dysfunction in Acute Sroke by Auricular Acupuncture Mediated by ABVN: A Clinical Study [Trial registry record]. Available online: https://trialsearch.who.int/Trial2.aspx?TrialID=ChiCTR-IPR-17011893 (accessed on 29 May 2023).

14. Jain, A.K.; Subhash, C.M.; Bhola, S.V.; Kushal, M.; Ashwini, M.; Jitendrapal, S.S. Effect of Yoga Lifestyle in Patients with Heart Failure: A Randomized Control Trial. Int. J. Yoga 2022, 15, 40–44. https://doi.org/10.4103/ijoy.ijoy_183_21. PMID: 35444368.
15. 1. Redwine, L.S.; Pung, M.A.; Wilson, K.; Bangen, K.J.; Delano-Wood, L.; Hurwitz, B. An exploratory randomized sub-study of light-to-moderate intensity exercise on cognitive function, depression symptoms and inflammation in older adults with heart failure. J. Psychosom. Res. 2020, 128, 109883. https://doi.org/10.1016/j.jpsychores.2019.109883. PMID: 31786338.
16. Yu, L.C.; Lin, I.M.; Fan, S.Y.; Chien, C.L.; Lin, T.H. One-Year Cardiovascular Prognosis of the Randomized, Controlled, Short-Term Heart Rate Variability Biofeedback Among Patients with Coronary Artery Disease. Int. J. Behav. Med. 2018, 25, 271–282. https://doi.org/10.1007/s12529-017-9707-7. PMID: 29297147.
17. Limmer, A.; Laser, M.; Schutz, A. Mobile Heart Rate Variability Biofeedback as a Complementary Intervention After Myocardial Infarction: A Randomized Controlled Study. Int. J. Behav. Med. 2022, 29, 230–239. https://doi.org/10.1007/s12529-021-10000-6. PMID: 34008159.
18. Del Pozo, J.M.; Gevirtz, R.N.; Scher, B.; Guarneri, E. Biofeedback treatment increases heart rate variability in patients with known coronary artery disease. Am. Heart J. 2004, 147, E11. https://doi.org/10.1016/j.ahj.2003.08.013. PMID: 14999213.
19. Krishna, B.H.; Pal, P.; Pal, G.K.; Balachander, J.; Jayasettiaseelon, E.; Sreekanth, Y.; Sridhar, M.G.; Gaur, G.S. Effect of yoga therapy on heart rate, blood pressure and cardiac autonomic function in heart failure. J. Clin. Diagn. Res. 2014, 8, 14–16. https://doi.org/10.7860/JCDR/2014/7844.3983. PMID: 24596712.
20. Chan W, Tsang WW. Short-term Tai Chi Training May Benefit Arterial Compliance But Not Heart Rate Variability Among Stroke Survivors: A Randomized Controlled Trial. The Journal of the International Society of Chinese Health Practices. 2020;1(1). Available online: http://www.ischp.org/ojs/index.php/jischp/article/view/4 (accessed on 29 May 2023).
21. Lin, I.M.; Fan, S.Y.; Lu, H.C.; Lin, T.H.; Chu, C.S.; Kuo, H.F.; Lee, C.S.; Lu, Y.H. Randomized controlled trial of heart rate variability biofeedback in cardiac autonomic and hostility among patients with coronary artery disease. Behav. Res. Ther. 2015, 70, 38–46. https://doi.org/10.1016/j.brat.2015.05.001. PMID: 25978746.
22. Lopes, C.P. Yôga e o treinamento de técnicas respiratórias em pacientes com insuficiência cardíaca com fração de ejeção preservada: Ensaio clínico randomizado. Doctoral Thesis, Universidade Federal do Rio Grande do Sul. Porto Alegre, Rio Grande do Sul, Brazil, 2017.
23. Lopes, C.P.; Danzmann, L.C.; Moraes, R.S.; Vieira, P.J.C.; Meurer, F.F.; Soares, D.S.; Chiappa, G.; Guimaraes, L.S.P.; Leitao, S.A.T.; Ribeiro, J.P.; et al. Yoga and breathing technique training in patients with heart failure and preserved ejection fraction: Study protocol for a randomized clinical trial. Trials 2018, 19, 405. https://doi.org/10.1186/s13063-018-2802-5. PMID: 30055633.
